# Second Gamma Knife Treatment for Trigeminal Neuralgia: Anterior Target Spacing and 25 Gy as the Second Dose

**DOI:** 10.7759/cureus.30761

**Published:** 2022-10-27

**Authors:** Bianca S Marquez, Ashley Nguyen, Sammie Coy, Beatriz Amendola, Aizik L Wolf

**Affiliations:** 1 Department of Neurosurgery, Miami Neuroscience Center at Larkin, South Miami, USA; 2 Surgery, Albert Einstein Medical Center Philadelphia, Philadelphia, USA; 3 Radiation Oncology, Innovative Cancer Institute, South Miami, USA

**Keywords:** ct cisternogram, mri, pain, postoperative numbness, balloon compression, microvascular decompression, type 1, stereotactic radiosurgery, gamma knife, trigeminal neuralgia

## Abstract

Objective

Gamma Knife® radiosurgery (GKRS) has been demonstrated to be a well-known approach for treating patients with medical refractory trigeminal neuralgia (TN). Herein, the authors review the outcomes of pain among a large cohort of patients who had undergone a second GKRS delivered at a significantly reduced dose.

Methods

The authors conducted a prospective analysis of patients who have undergone two GKRS procedures between the years 2012 to 2021 at one institution. Baseline characteristics, radiosurgical dosimetry and technique, pain outcomes, and adverse effects were reviewed. Pain outcomes were measured with the Barrow Neurological Institute (BNI) pain intensity scale, which included the best BNI attained after the last treatment and recurrence.

Results

A total of 202 patients were identified, including 55 males and 147 females. Pain recurrence was reported in all patients prior to the second GKRS treatment (median = 4 months). Pain recurrence in the preceding Japan Neuroscience Society (JNS) 2021 study was also reported in all patients after each GKRS with a median value of 20 months between the second and third procedures. Complete to partial pain relief (BNI ≤ III) was achieved in 80% of patients after the second treatment. Over a median of 12 months of follow-up, 60% of patients maintained complete to partial pain relief compared to 77% of patients over the course of three treatments. In the present study, one patient developed facial spasms while 10 patients experienced persistent facial tingling. Subjective mild numbness was also found to be present in 16% of patients, with only 2% being bothersome, as compared to the JNS study, where subjective mild numbness was found to be present in 14%, with only 14.3% being bothersome. Among the 202 patients, 74 (37%) patients had undergone subsequent additional procedures such as a third GKRS, microvascular decompression (MVD), or other percutaneous procedures.

Conclusion

The authors describe the largest study to date of patients undergoing a second GKRS treatment for type 1 medical refractory trigeminal neuralgia. A reduced dose of radiation for a second treatment may produce outcomes similar to those of three consecutive treatments in regard to limiting recurrence and adverse effects.

## Introduction

Trigeminal neuralgia (TN) is a rare facial pain disorder characterized by severe unilateral “electric shock-like” pain in one or more divisions of the trigeminal nerve. Type I TN is characterized by episodic attacks while type II TN is characterized by persistent facial pain [1]. While a wide variety of medical and surgical treatments have been developed to treat TN, the recurrent nature of the disease makes long-term relief a challenge.

Gamma Knife® radiosurgery (GKRS) is a well-studied, noninvasive treatment modality for medically refractory and high-risk surgical TN patients [2-4]. Like many treatment options, GKRS provides excellent pain relief but fewer permanent complications [5]. Patients who experience pain relapse with a previous favorable response to an initial GKRS can be selected for a second GKRS treatment [6]. Evidence from prior studies indicates that a second GKRS treatment may give sustained pain relief but also results in increased toxicity, often leading to facial numbness [6-8].

In the present study, we determine the clinical outcome of patients with type 1 TN that underwent two GKRS treatments, which accounts for the largest study to date. Additionally, we explore the significance of decreasing the dosage of the second GKRS treatment on pain relief durability and toxicity.

## Materials and methods

Patient population

We performed a prospective review of patients who had undergone two GKRS procedures for type 1 medically refractory TN between the years 2012 and 2021 at the Miami Neuroscience Center at Larkin Community Hospital. An initial cohort of 226 patients was identified using electronic health records. We excluded patients with incomplete records (n = 1) and type 2 trigeminal neuralgia (n = 23). The final cohort comprised 202 patients. All patient interactions were reviewed from documentation within the electronic health records. Data pertaining to demographics, past medical history, radiosurgical technique, age at second treatment, pertinent procedures before the initial GKRS, pertinent past medical history, pain outcomes, and postoperative adverse effects were all collected from the electronic health records. This study was approved by the Larkin Community Hospital International Review Board (LCH IRB) (F-0822CSW).

Radiosurgical Procedures

GKRS treatments were performed on a Leksell model G by a team consisting of a neurosurgeon and a medical physicist. The head frame was placed by the neurosurgeon under local anesthesia. Dose selection and anterior target shift were performed on Leksell computerized software. A max initial dose of typically 38-40 Gy at least 1 mm anterior to the 50% isodose line within the dorsal root entry zone was delivered for the first treatment. A max dose of 25 Gy was delivered for the second treatment. The volumes for each procedure were calculated from the computerized software. The 50% isodose line was typically tangential to the brainstem for all patients. Imaging with MRI or CT cisternogram was performed using axial sequences.

Statistical Analysis

Data collection was performed using Microsoft Excel (Microsoft Corporation, Redmond, WA). Descriptive statistics pertaining to patient baseline characteristics, radiosurgical parameters, and treatment outcomes were categorized into tables. Pain intensity after the second GKRS procedure and at the last follow-up after the second GKRS was categorized using the Barrow Neurological Institute (BNI) scale [9]. Complete pain relief with no medication required was defined as a score of I. Occasional pain was defined as a score of II if no medication was required and III if the pain was controlled with medication. Some pain, not adequately controlled with medication, was defined as a score of IV. No pain relief or severe pain was defined as a score of V. Treatment failure after the second GKRS treatment was defined by pain recurrence with a BNI score of IV or V at the last follow-up.

Postoperative adverse effects were categorized into tables. The adverse outcomes were grouped according to symptoms, including facial tingling, spasms, and numbness. Facial numbness was grouped into two categories: mild and bothersome. All adverse effects were counted as subjective in nature because the symptoms were mostly patient-declared.

No statistical tests determining statistical significance were performed. Descriptive statistics pertaining to baseline characteristics and treatment outcomes were tabulated as frequency. Categorical (age) and continuous (duration) data were tabulated as a median with a corresponding interquartile range. Dosimetric data were performed in the same manner.

## Results

Patient demographics

Demographic and baseline characteristics are shown in Table 1. The median age at the time of the second GKRS was 70 years. Our sample contained 55 males and 147 females. Prior to GKRS procedures, all patients had well-described recurrent episodic pain in one or more distributions of the face. Seven out of the 202 developed bilateral symptoms. One patient had a past medical history of herpes simplex virus while two other patients had a history of herpes zoster. Five patients (2%) had a past medical history of multiple sclerosis. Before the first GKRS, 35 patients (17%) had undergone at least one surgical therapeutic modality, including seven microvascular decompressions (MVDs) and 28 percutaneous procedures such as rhizotomy or balloon compression.

**Table 1:** Baseline Characteristics PMHx = past medical history, MVD = microvascular decompression, HSV = herpes simplex virus, MS = multiple sclerosis

Variable	Value
Median age at 2nd GKRS in years (IQR)	70 (59-76.75)
Male	55 (27%)
Female	147 (73%)
Type 1	202 (100%)
Right	123 (61%)
Left	79 (39%)
Bilateral symptoms	7 (3%)
Prior MVD	7 (3%)
Prior dental	23 (11%)
Prior peripheral nerve blocks/neurectomies	21 (11%)
Prior combinations	6 (3%)
Prior balloon compressions	7 (3%)
MVD b/w GKRS	8 (4%)
No previous procedures	151 (75%)
HSV/HZV	3 (1%)
MS	5 (2%)

Pain Relief Outcomes

Using the BNI scoring method, complete to partial pain relief was achieved in 80% of the patients, and 60% maintained long-term relief. Repeat treatments (e.g., additional GKRS) were done when the pain returned, worsened, or remained unchanged from the second GKRS with BNI scores of IV or V. The time intervals (in months) were reported as both a range and a median value for the following: pain recurrence, between the first and second procedure, and the last follow-up. The results are summarized in Table 2.

**Table 2:** Pain Relief Scores and Adverse Effects BNI = Barrow Neurologic Institute pain score, FU = follow-up, MVD = microvascular decompression

Variable	Value
Best BNI: I	5 (2%)
Best BNI: II	1 (0.5%)
Best BNI: III	155 (77%)
Best BNI: IV	18 (9%)
Best BNI: V	23 (11%)
FU BNI: I	3 (1%)
FU BNI: II	1 (0.5%)
FU BNI: III	117 (58%)
FU BNI: IV	33 (16%)
FU BNI: V	48 (24%)
Time to pain recurrence in months Median (IQR)	4 (1 - 9)
Time between the two GKRS in months Median (IQR)	15 (6-33)
Time to last follow-up in months Median (IQR)	12 (10.5-36)
Subjective Mild facial numbness	32 (16%)
Bothersome facial numbness	5 (2%)
Facial tingling	10 (5%)
3rd GKRS	11 (5%)
MVD	42 (21%)
Balloon Compression	18 (9%)
Botox Injection	1 (0.5%)
Glycerol Injection	0 (0%)
Nerve Block	1 (0.5%)
Peripheral Neurectomy	1 (0.5%)

Toxicity

The most common adverse effects were mild subjective facial numbness, spasms, and tingling. Subjective mild numbness was found to be present in 16% of patients, with only 2% reporting the symptoms as bothersome. Three patients with bothersome numbness achieved and maintained partial alleviation of pain (BNI of III) while two patients in this category failed to achieve or maintain pain alleviation (BNI of IV and V, respectively). One patient experienced spasms and 12 patients experienced tingling. No patients experienced anesthesia dolorosa.

Dosimetry

**Figure 1:**
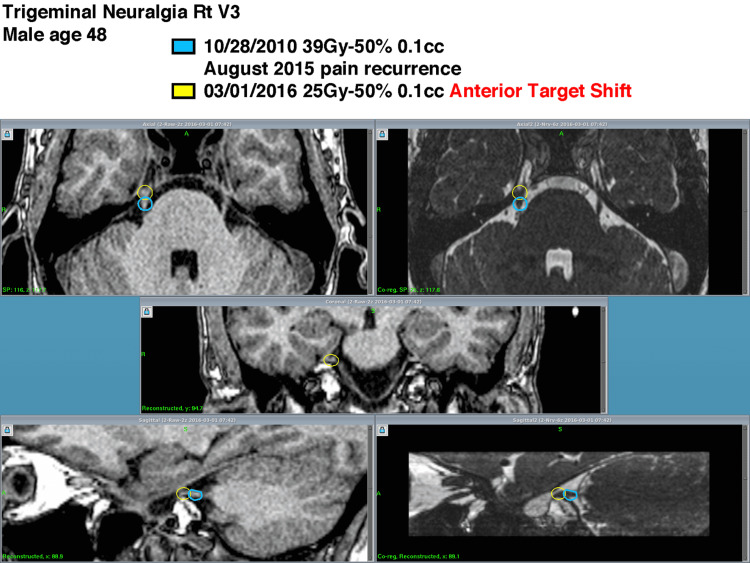
Example Outlining the Dosimetric Plan of Two GKRS Procedures to the Right Trigeminal Nerve GKRS = Gamma Knife® radiosurgery

Dosimetric data are depicted in Table 3 with an illustrated example in Figure 1. The median dose delivered at the first procedure was 40 Gy and the median dose delivered at the second procedure was 25 Gy. Both the median volumes for each treatment were calculated to be 0.1 and the total value calculated for the anterior target shift was 0.814.

**Table 3:** Dosimetric and Radiosurgical Data MRI = magnetic resonance imaging, CT = computerized tomography

Variable	Value
Median dose at 1st GKRS in Gy (IQR)	38 (38-39)
Median dose at 2nd GKRS in Gy (IQR)	25 (25-25)
Treatment Planning: MRI	183 (91%)
Treatment Planning: CT w/ cisternography	17 (8%)
Treatment Planning: Both	2 (1%)
Median anterior target shift (IQR)	0.814 (0.814-0.814)
Median Volume: 1^st^ GKRS	0.1 (0.9-0.1)
Median Volume: 2^nd^ GKRS	0.1 (0.9-0.1)

Additional Procedures

A subset of patients underwent additional procedures such as third GKRS (5%), MVD (21%), balloon compression (9%), or other percutaneous procedures (0.5%). All these patients did not achieve resolution of pain following the second GKRS. As a result, they were offered, or sought out, other methods of pain relief. The patients (42) who underwent an MVD have, to date, not had any future relapses in pain.

## Discussion

Despite a wide, diverse selection of medical and surgical management having been well-established, long-term relief remains a challenge because of the recurrent nature of the disease. Evidence from Burchiel et al. suggests a simple framework for understanding and categorizing TN. Type 1 TN is defined as pain that is episodic while type 2 is defined as pain that is consistently greater than 50% [5,9]. First-line medications include the administration of either carbamazepine or oxcarbazepine. Second-line medications used for patients unresponsive to first-line agents include phenytoin, pregabalin, gabapentin, or tricyclic antidepressants. Traditional surgical management involves performing microvascular decompression. While MVDs may have had high rates of resolution of pain postoperatively (80-98%), Lieu et al. report approximately a 10-30% rate of relapse [10,11]. In a systematic review of indicated treatment modalities for TN, Lambru et al. developed an algorithm for appropriate management based on the type and image-based evidence showing neural compression [12]. Sharma et al. advised MVD (96%) as the best surgical management for type 1 TN while de-emphasizing the use of radiosurgery (71.8%); relative risk of 1.309 (95% CI 1.217-1.409; P <0.001) [13]. While this study undermines radiosurgery as a viable technique, we consider this to be due to the author's interpretation of the limited evidence presented at the time of publication. Collectively, these two reviews emphasize MVDs' relative success in patients with clear neurovascular contact and validate the need for Gamma Knife radiosurgery data on pain relief for resistant cases of TN.

Evidence from two recently published works from 2020 and 2021 on the results of having three Gamma Knife procedures suggest that a third procedure reduces relapses and produces comparable effects for long-term pain relief in at least 77% of patients when compared to the initial or second treatments, respectively [14,15]. Higher doses of radiation manifest as axonal damage and edema at higher doses, yet post-radiosurgery inflammation is not evident [14-18]. Both studies predominantly focus on the outcomes of pain relief after three GKRS treatments with incrementally higher doses per repeat treatment. Thus, three or more Gamma Knife treatments may suggest an increased susceptibility to the development of adverse postoperative effects as a result of axonal edema [11,19]. While repeat GKRS has been widely analyzed, to date, no published works have reported the long-term effects or outcomes of a second GKRS with an accompanied dose of at least 25 Gy but less than 40 Gy [11].

Combined findings in the two studies on outcomes of pain relief involving three or more GKRS in a heterogeneous group of patients are subject to the fundamental limitations of small sample size and study design. Our results are thus comparable to studies showing improved and controlled pain relief in patients having at least three procedures (93% and 77%; respectively). We once again highlight the safety and efficacy of having a second GKRS without the need for subsequent treatments in patients with type 1 TN. Herein, lies future opportunities for emerging studies on GKRS effectiveness for recurrence for the type 1 TN population.

This study is subject to the intrinsic limitations of a small sample size and study design. Although this is considered the largest study to date, the reduced sample size precludes the power to detect differences in postoperative outcomes. The single institution and exclusion criteria inherently introduce a selection bias, subsequently limiting the study’s external validity.

## Conclusions

We introduce the largest prospective analysis of patients treated with a second GKRS with a reduced dose of radiation for recurrent type 1 TN. Our results suggest that limiting the treatment to two sessions and lessening the radiation is highly effective, with at least 80% of patients gaining complete to partial pain relief. Our results also suggest that in the long term, at least 60% of patients retain complete to partial relief for a median of 12 months. Subjective postoperative numbness was the most common adverse effect. Although all patients with reported mild numbness had concomitant complete or partial pain relief, additional studies are needed to verify the validity of this treatment strategy to other patient populations, specifically patients with type 2 TN.
